# TLSO with Graphene Sensors—An Application to Measurements of Corrective Forces in the Prototype of Intelligent Brace

**DOI:** 10.3390/s22114015

**Published:** 2022-05-25

**Authors:** Patrycja Tymińska, Katarzyna Zaborowska-Sapeta, Daniel Janczak, Tomasz Giżewski

**Affiliations:** 1 Lublin University of Technology, Nadbystrzycka Street 38D, 20-618 Lublin, Poland; 2 University of Warmia and Mazury in Olsztyn, Michała Oczapowskiego Street 2, 10-719 Olsztyn, Poland; katarzyna.zaborowska@uwm.edu.pl; 3 Warsaw University of Technology, Plac Politechniki 1, 00-661 Warsaw, Poland; daniel.janczak@pw.edu.pl

**Keywords:** sensor, graphene, brace, scoliosis, pressure force

## Abstract

This study presents a prototype of Intelligent Brace—the gold medal winner in the 68th edition of the International EUREKA 2019 Competition, in Valencia, Spain. It shows how to improve the effectiveness of a static orthopedic brace, with modern technology based on advanced electronic solutions. The research uses in-house-made thin-film graphene sensors, an electronic system with a microcontroller and derotating pads, a mobile application for Android system, and a remote database. The article presents a description of the real project, the system principle of operation, and the layout of the system elements in the orthosis. A prototype device was designed and built that was tested not only in laboratory conditions, but also during trials with the first patient. Approximately two months of data were collected and interpreted. The collected research results provided basic knowledge about the range of forces exerted by the brace on the patient’s body, as well as the regularity of wearing the orthosis by the patient and compliance with the doctor’s recommendations.

## 1. Introduction

The Cheneau brace is an orthosis that corrects three-plane deformation of the spine, by exerting multi-point pressure forces on the torso. In the frontal plane, the brace exerts forces displacing the spine and the thorax in the direction of the body axis, and in the transverse plane it derotates forces. In the sagittal plane, the brace’s action is directed at reconstruction of the physiological curvature of the spine. Special pressure-relieving spaces designed in the brace enable the spine and the chest to move in the desired direction, under the influence of exerted forces. The indication for treatment with a Cheneau brace is confirmed progressive idiopathic scoliosis (SI), with a curve angle measured on a Cobb radiograph greater than 25° [1]. A good therapeutic effect is determined by the proper qualification, the proper fabrication of the orthosis, and, above all, the patient’s compliance with the therapeutic protocol [1]. The correction of the curve angle in a brace is, inextricably, linked to the pressure forces exerted by the brace on the torso, in a multi-point system. The measurement of pressure forces allows to control the brace’s fit. The comparison of the measurement results, with the corrective effect determined on an X-ray performed while in the brace, enables optimization of treatment, by determining the maximum effective pressure force causing displacement of the spine column in the desired direction. A complex data collection and acquisition system, where thin-film pressure graphene sensors are integrated with an environment for processing and visualization of data-ready interpretation and evaluation by a physician, was designed in order to improve the idiopathic scoliosis therapy process and shorten its time.

## 2. Relevant Research

There are similar solutions of pressure force measurement in the Cheneau brace, described in the literature, but they always differ from each other. In the article by Lou et al. [2], pressure forces are measured using a commercial sensor, the SENSYM FS01, which is distinguished by its low power consumption, small size, and low purchase cost. It works in the force range of 0 up to 6.82 N. The device, also, has a system for wireless data transmission, at a maximum distance of 30 m, and data storage in the internal memory of the microcontroller [3]. Data are collected at a frequency of 1 record per minute (0.0167 Hz), for a period of two weeks. The average force values were 1.52 ± 0.75 N. The same authors, also, presented an alternative way of controlling the wearing and measuring of the forces exerted by the orthosis on the patient’s body. They used an additional airbag module, with the purpose of maintaining the required pressure force. When the orthosis does not apply enough pressure, the cushions are pumped to provide such pressure and, thus, increase the effectiveness of treatment for patients with scoliosis [4]. In the solution described by O. Dehzagi et al. [5,6], an additional element used in the system is an accelerometer with a gyroscope, which determines the patient’s body position. The pressure forces are measured using Honeywell FSB1500NSB sensors. Data is stored at 40 Hz, on an included microSD memory card, and transmitted wirelessly via Bluetooth. The forces applied in this brace reach values of 300 N up to 500 N. The two single sensors used in this project perform point force measurement, which, according to the authors, is very desirable. The research described in this paper included measurements in specific body positions, in order to create a universal force distribution model for the treatment of scoliosis [7]. Bansode et al. [8] presented a solution, in which the applied pressure sensors were used for a different purpose than in previous inventions. Namely, the orthopedic brace was equipped with airbags, inside which MPX5010GP pressure sensors were placed, with the purpose of measuring the pressure in the aforementioned airbags. The information about the pressure was sent to a microcontroller, so that the level of filling the airbags with air was changed and adjusted to the needs, using an appropriate algorithm. A 16 × 2 LCD display was, also, used for continuous monitoring of the pressure values in the brace. The article by Hudák et al. [9] presents another approach to optimizing the treatment of idiopathic scoliosis, with the use of an orthopedic brace. In their design, instead of individual sensors they use a Pressurex mat that measures pressure over its entire surface. Its thickness is 0.1–0.2 mm. Measurements were taken at a frequency of 49 Hz. The mat changes color under pressure, the higher the pressure is, the more intense the color. The system also has a data storage system, which allows the doctor to monitor the value of the brace’s pressure throughout the course of treatment. A project on the Cheneau brace, presented by Evans et al. [10], has pressure sensors measuring the pressure of the brace on the patient’s body as well as temperature and humidity sensors, which help to determine how long the brace has been worn. The pressure measurement is performed continuously, while the wearing time is counted only when the appropriate temperature is registered in a predetermined range, and the humidity level is recorded when the brace is in contact with the patient’s skin. Typically achieved force values were in the range of 0 N up to 5.1 N. They were measured with commercial sensors FS01 and FSS1500N from Honeywell International Inc., Charlotte, NC, USA. The design of their own pressure sensors, which are supposed to be imperceptible by the orthosis wearer, with a low cost of production, is, also, presented. They described two main approaches, one using a rubber substrate and the other using an inelastic polymer. They measured forces in the range 0 N up to 15 N. Fuss et al. [11] developed a cost-effective and accurate pressure sensor system for TLSOs. In their paper, they present the study of a piezoresistive polymer sensor placed between two foam liners. The sensors were tested underneath a chest belt and inside the scoliosis brace, during different activities, and proved to react to the changing pressure during those activities. The measured pressure in the brace was 0.014–0.124 MPa. The research shows that the foam padding inside the scoliosis brace causes the pressure-conductance curve to be affected by hysteresis.

## 3. Materials and Methods

### 3.1. Thin-Film Pressure Sensors

**Figure 1:**
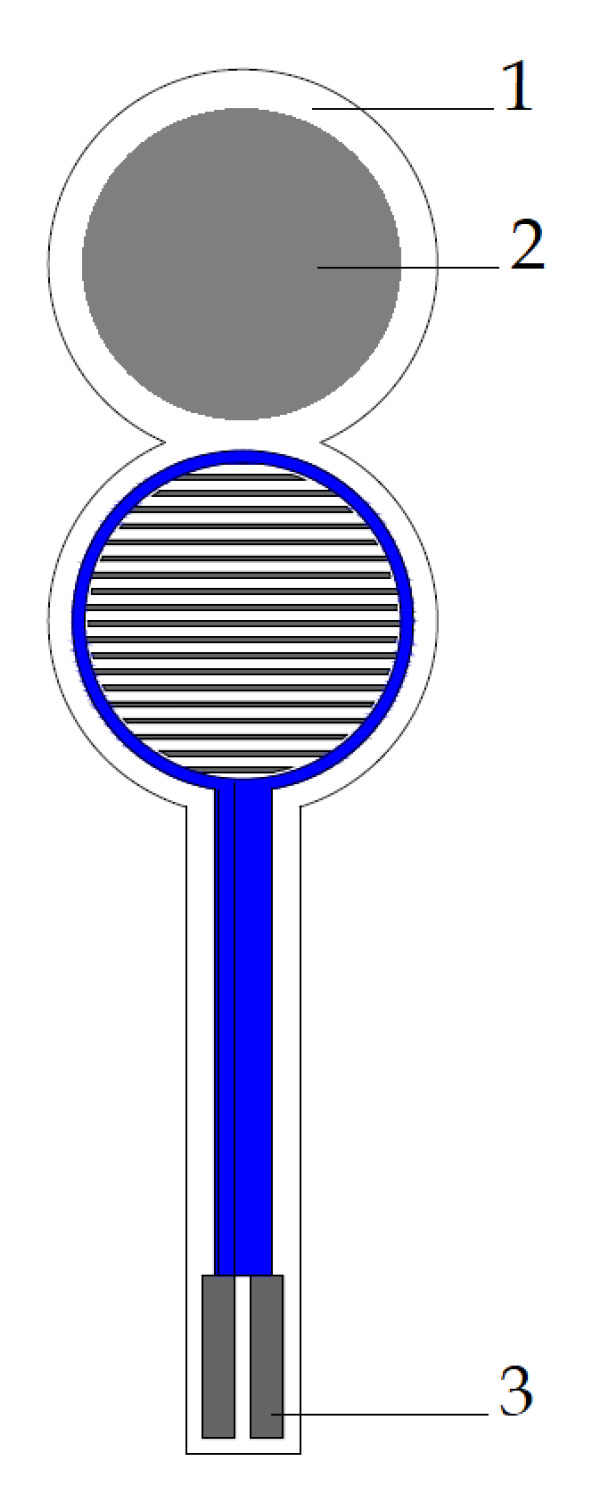
Sensors used in the system: (**1**) polyethylene terephthalate (PET) film, (**2**) graphene layer, and (**3**) silver layer–comb electrode.

**Figure 2:**
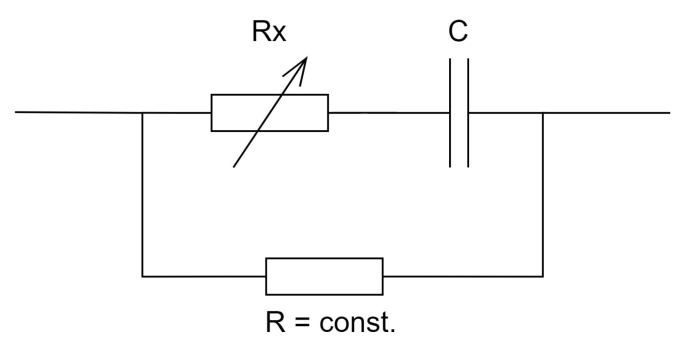
Equivalent circuit of the sensor.

In order to study the distribution of pressure forces in the brace, thin-film sensors (Figure 1) were used, consisting of two main elements: a silver comb electrode and a graphene circle that forms the active sensing surface [12]. The silver conductive paths bring the signal to the active area, where they branch into a network of narrow, parallel electrodes that forms an interlocking comb layout. Under the influence of pressure, the contact resistance in the graphene layer changes, which results in a change in the sensor’s impedance parameters [13], which are converted into the value of the pressure force. The sensors allow to detect even the smallest values of the pressure force. Figure 2 shows an equivalent circuit of the thin-film graphene sensors.

The calibration and classification of the sensors was carried out, using a mechanical system that allowed to exert a pressure force of 0 N to 450 N to the sensor, which was carried out in two stages. First, a preliminary classification was made on the basis of load tests with DC power (according to the brace application, where battery power was used), which was, then, repeated for the sensors with the highest accuracy with sinusoidal alternating current, in the frequency range from 40 Hz to 2 MHz.

**Figure 3:**
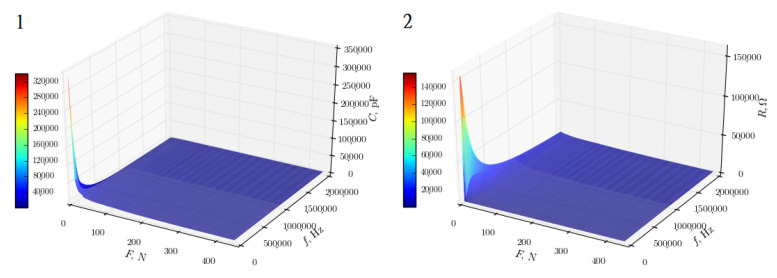
Impedance characteristics of the highest quality sensor: (**1**) capacitive and (**2**) resistive [14].

**Figure 4:**
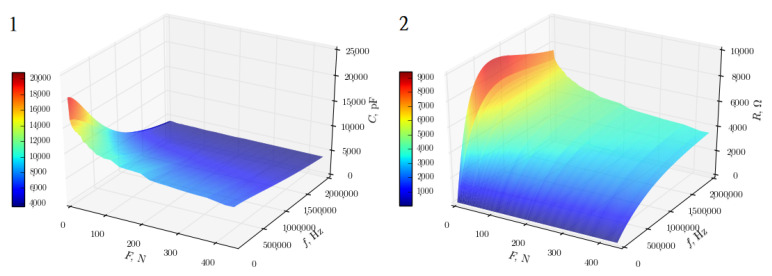
Impedance characteristics of the defective sensor: (**1**) capacitive and (**2**) resistive [14].

The research with sinusoidal AC of variable frequency allowed for a deeper understanding of the nature of the sensors—on the basis of the research it can be concluded that they are not purely resistive sensors, but impedance sensors with a significant share of the capacitive component. The impedance analysis, also, allowed to select sensors with the best impedance characteristics, which were used in the brace, and to detect defective ones. In a correctly made sensor (Figure 3), both resistive and capacitive characteristics are, exponentially, decreasing in the full spectrum of the tested frequencies. The degree of slope of the curve indicates the degree of sensitivity of the sensor to changes in the pressure force value. In the case of a defective sensor, there is a local extreme in the resistive characteristic (the red area in Figure 4), and the capacitive characteristic is not as sloped as in the case of a normal sensor. This indicates an incorrect design and a low sensitivity of the tested sensor, since, due to the sponge-comb structure of the sensor, its resistance should not increase with increasing frequency of the current.

**Figure 5:**
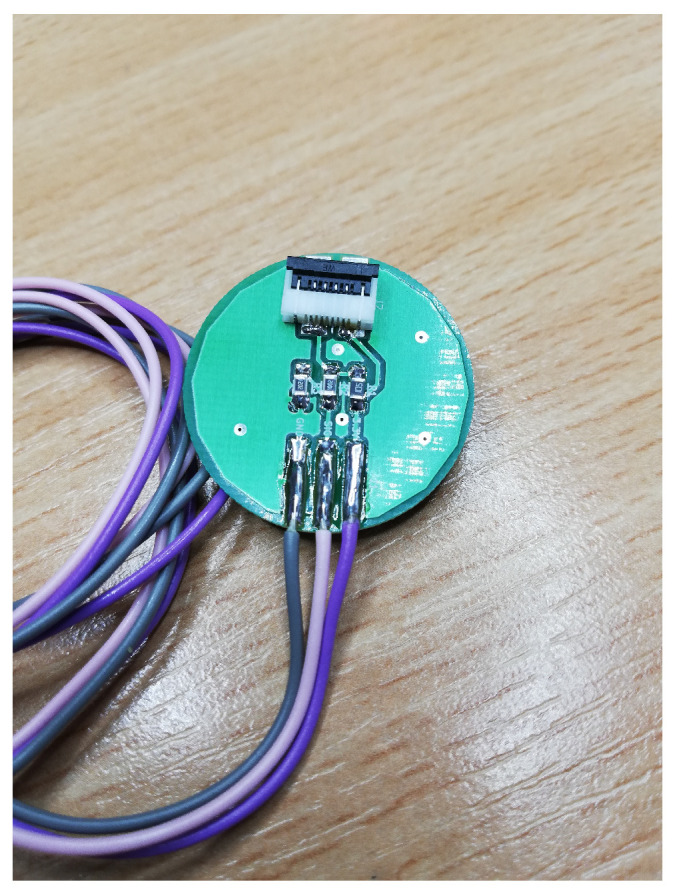
Electronic circuit designed to enable connection between the microcontroller and the pressure sensor.

**Figure 6:**
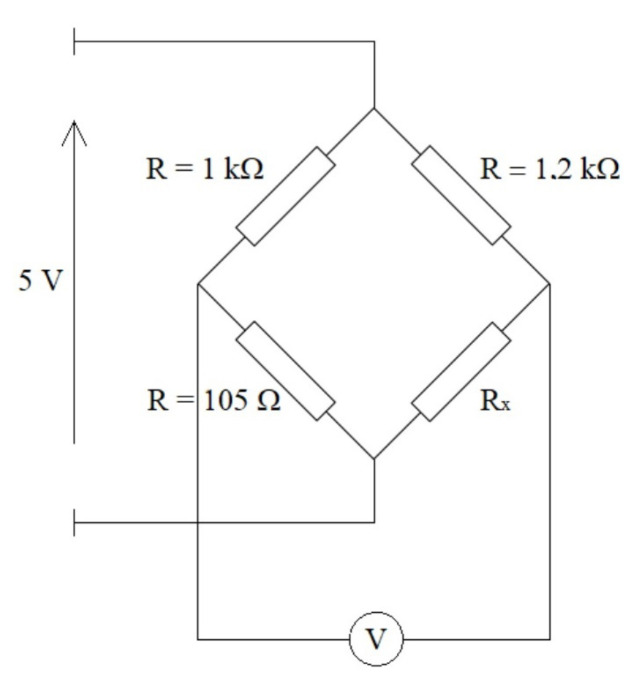
Diagram of the pressure-sensor measurement system.

In order for the microcontroller to correctly aquire data from the sensors, an additional measuring system had to be designed. The system supplies power to the sensors and outputs a voltage signal from the sensor (Figure 5). The PCB board has a connector for a sensor, a measuring system, and outputs that allow connection to a microcontroller. The measurement system operates on the principle of a Wheatstone bridge (Figure 6). It consists of four branches, formed by four resistors and a power source. One of the resistors is the pressure sensor. The sensor in the brace, depending on pressure, changes its resistance, and the resistance is measured by the microcontroller. The use of a bridge allows to increase the accuracy of measurements, by reducing the influence of temperature on the measurement as well as transforming the changing resistance into a changing voltage, interpreted by the microcontroller.

This type of bridge is, usually, used to measure small and rapidly changing resistances in DC circuits. The measurements are based on measuring the voltage at the output from the bridge and, then, determining the measured resistance. The accuracy of the measurement depends on the accuracy of the individual electronic elements creating the bridge—accuracy of resistors and a voltmeter measuring the output voltage [15].

### 3.2. System

**Figure 7:**
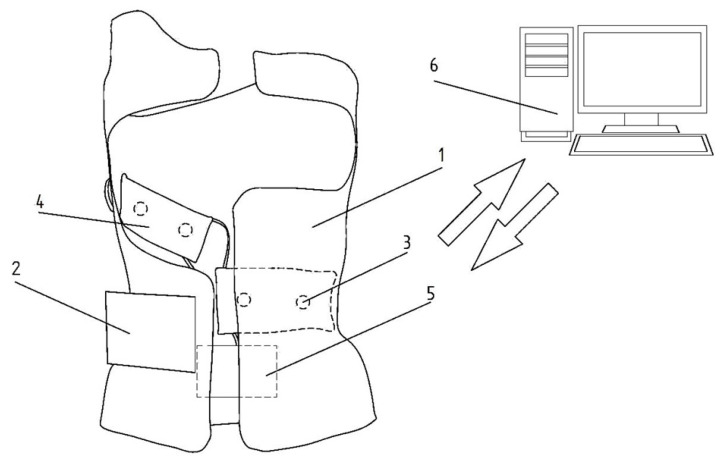
Front view of the rehabilitation brace: (**1**) rigid brace, (**2**) microcontroller, (**3**) pressure sensors, (**4**) pneumatic cushion (derotation airpads), (**5**) pump, and (**6**) control device (database).

**Figure 8:**
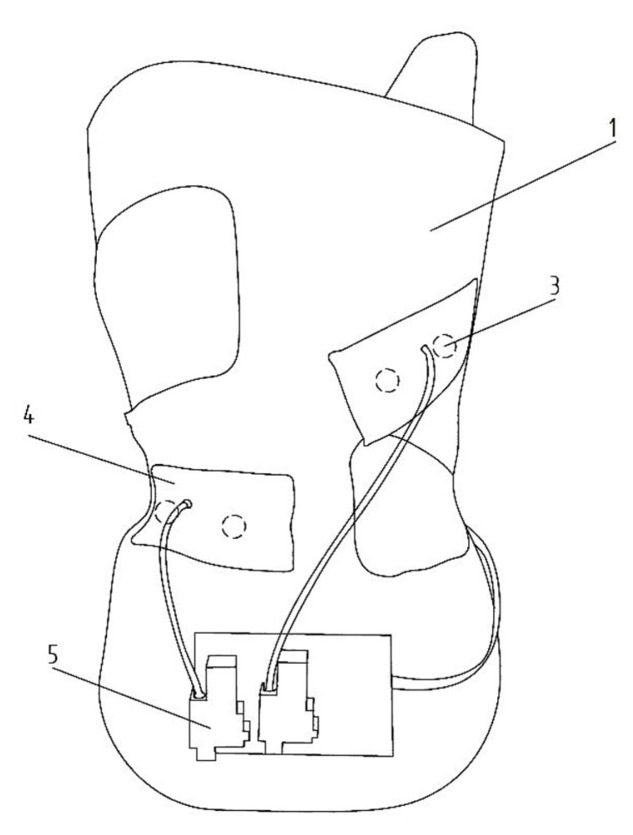
Rear view of rehabilitation brace: (**1**) rigid brace, (**3**) pressure sensors, (**4**) pneumatic cushion (derotation airpads), and (**5**) pump.

**Figure 9:**
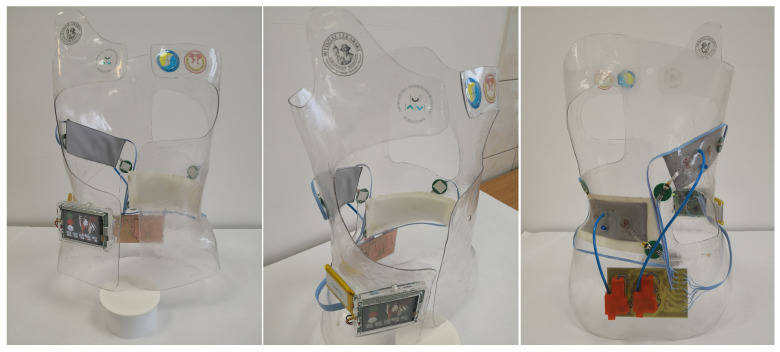
Prototype of a smart Cheneau brace.

The use of an autonomous system, providing measurement of the effective force exerted by the derotating pads in the brace and determination of the reference range of applied pressures depending on the patient’s bone age as well as the type of spinal curvature, is extremely important in the evaluation of both therapy and decision-making, in the treatment path of patients with scoliosis. It is, also, important to build a knowledge base, in order to create the fitting rules of a rigid orthosis, based on the measurement of the pressure dynamics in the brace and the doctor’s decision, as well as to control the therapy discipline. In order to solve those problems, a microprocessor-based supervision system (Figure 7 and Figure 8) has been developed, to collect data on the child’s posture using graphene sensors, visualize it on a 2.8${}^{\prime \prime }$ (320 × 240) TFT color display with a touchscreen, and adjust the level of the airbags filling in the regions of the derotation cushionsm to force the user of the brace to correct their posture. This was accomplished by the implementation of a portable system, based on mikromedia, for an STM32 M4 board with an STM32F407VGT6 microcontroller unit that provides real-time monitoring of the pressure forces that occur in the brace, collecting the data on a microSD card and sending it, via a GSM network, to a database located on a server. The microcontroller measures resistance using analog ports. The ADC module of the microcontroller converts the analog signal from the sensors into a digital signal, which is then converted into voltage and force. The data recording is carried out with the frequency of 1 measurement per second. The project also contains a pneumatic system, to control the filling of the derotating pads responsible for the pressure applied on the patient’s body, with a pump to control the inflation of the derotation airpads, which increase the pressure of the brace. The system is battery powered and can be charged with a USB cable. All elements are placed in the rigid orthosis, which is the main component of the therapeutic solution for idiopathic scoliosis—it exerts pressure on the patient’s body The use of the above-mentioned procedures allows the doctor to observe and determine the optimal pressure forces, thus increasing the corrective properties of the brace and increasing the effectiveness of the treatment method. The actual brace, with an installed microprocessor-based supervision system, is shown in Figure 9.

### 3.3. Database

A database, located on a server, is used to collect the data received from a Cheneau-type intelligent orthopedic brace. It stores information about the measurements received from thin-film graphene pressure sensors. Communication between the orthopedic brace and the database is carried out using two GSM modules. The first is installed in the brace, the second is installed in the desktop computer, where the database is located. Text messages are used to transmit the content, and the data exchange is bidirectional.

The applied pressure is to be selected on the basis of an algorithm, developed using a mathematical model. The pneumatic system used in the orthosis will be deactivated during the patient’s sleep, in order to ensure maximum comfort when using the device. It is very important to develop the right algorithm to maintain the correct pressure in the individual derotation pads, which is sufficient to ensure the most effective correction of the disease, while maintaining fair comfort. Therefore, the wireless communication between the server and the brace was given great attention in this project.

### 3.4. Application for Android System

**Figure 10:**
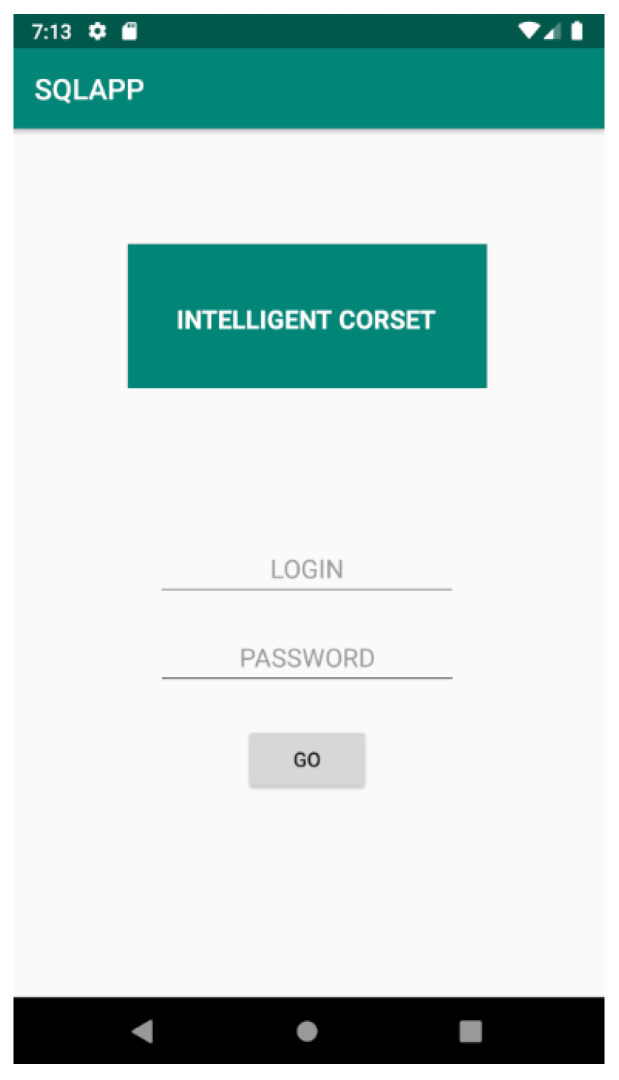
Cheneau smart brace application login screenshot.

Visualization of the pressure forces applied in the brace, in addition to the LCD in the device, is possible through an application for an Android phone. It is designed for the parents of the patient or the doctor treating them. The appearance of the application is shown below. First, there is user authorization, so only a limited group of people (parents, doctor, patient) have access to the data (Figure 10).

**Figure 11:**
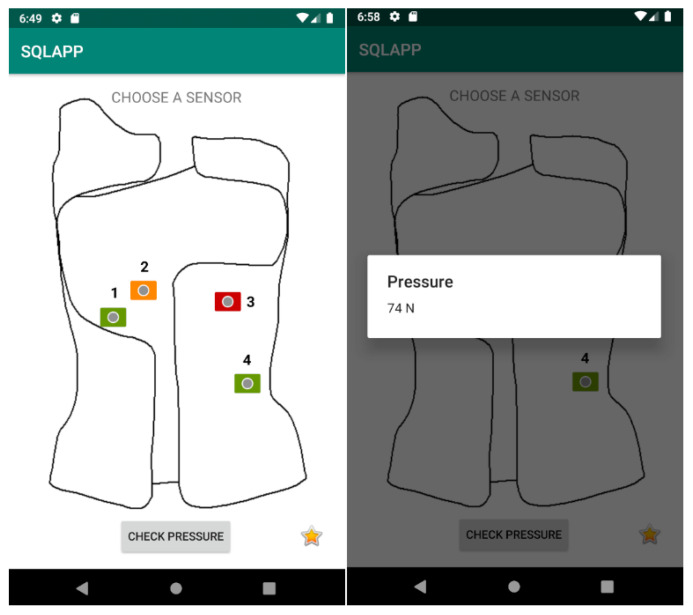
Screenshots of the Cheneau smart brace application, showing the current pressure on the sensor.

After correct authorization, a schematic drawing of the brace appears on the screen, with sensors marked in specific places. The colors of the sensors indicate the amount of pressure the brace applies on the body: green—the pressure is appropriate, orange—the pressure reaches the limit value, and red—the pressure is too low. By clicking on a particular sensor, it is possible to see the pressure at a given moment, in the form of a number (Figure 11).

**Figure 12:**
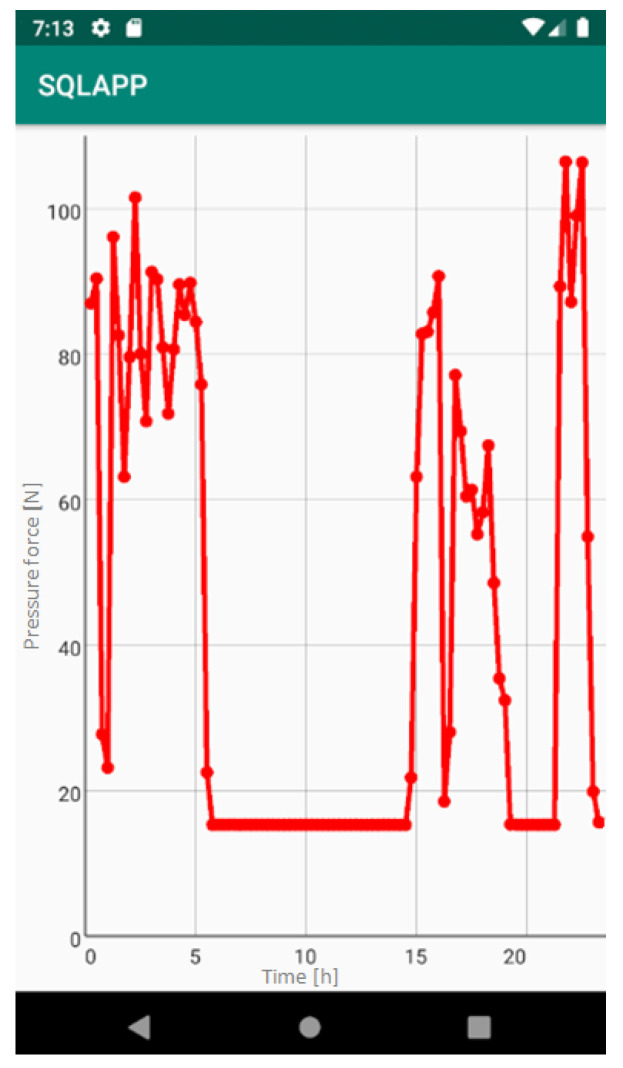
Screenshot of the Cheneau smart brace application, showing a graph of pressure force, from a given sensor over 24 h.

The application allows to display a graph showing the pressure force, for a given sensor in the last 24 h, by a long click on a specific sensor (Figure 12).

## 4. Results

**Figure 13:**
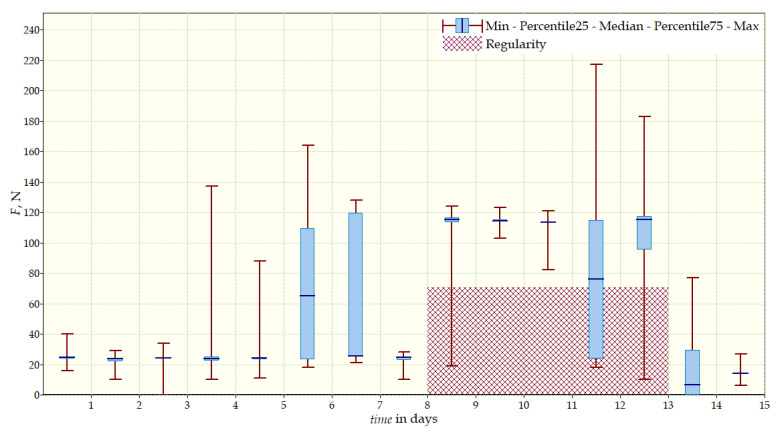
Measurement data from the first brace—sensor No. 1.

**Figure 14:**
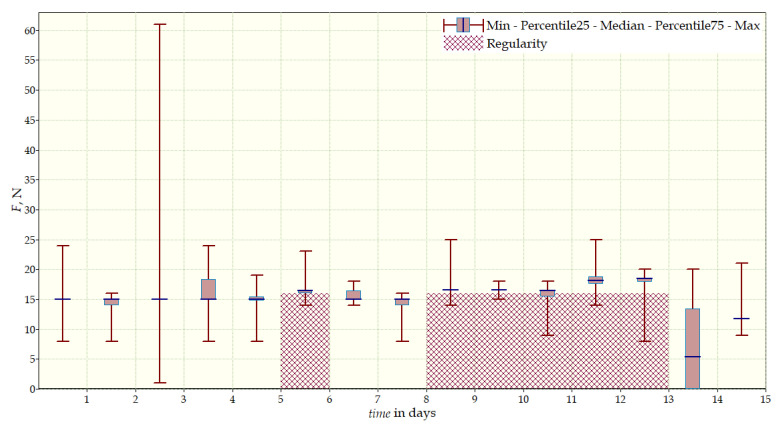
Measurement data from the first brace—sensor No. 2.

**Figure 15:**
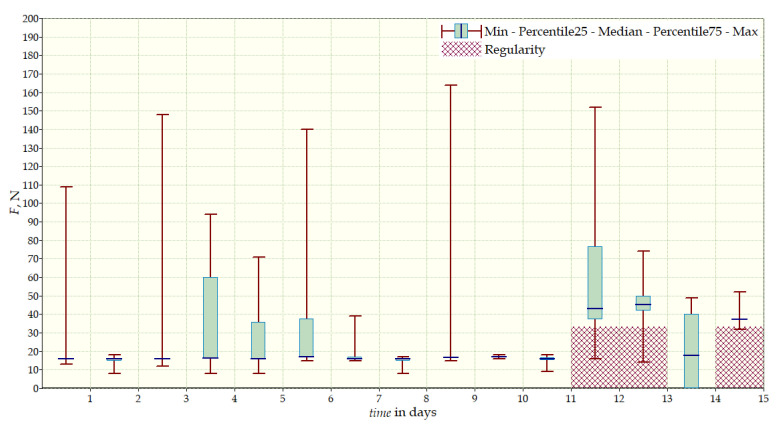
Measurement data from the first brace—sensor No. 3.

**Figure 16:**
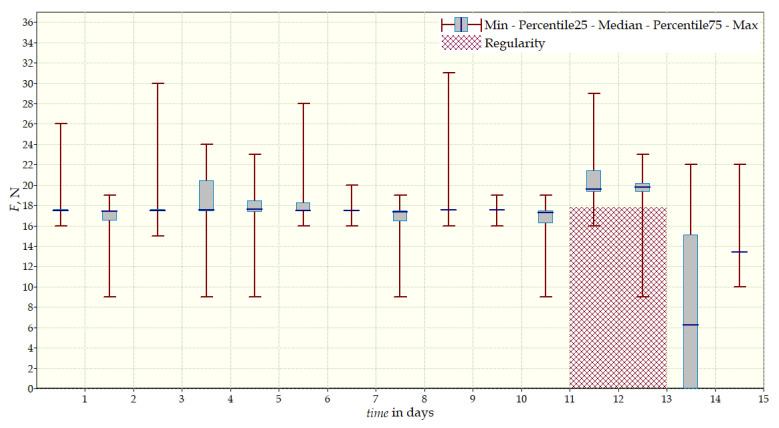
Measurement data from the first brace—sensor No. 4.

**Figure 17:**
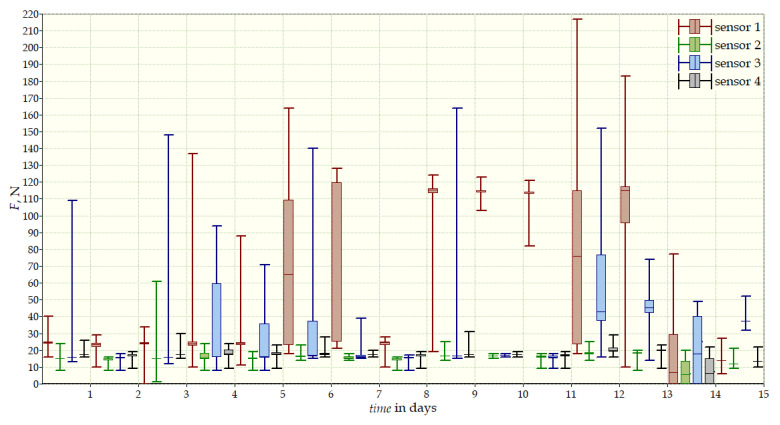
Measurement data from the first brace.

**Figure 18:**
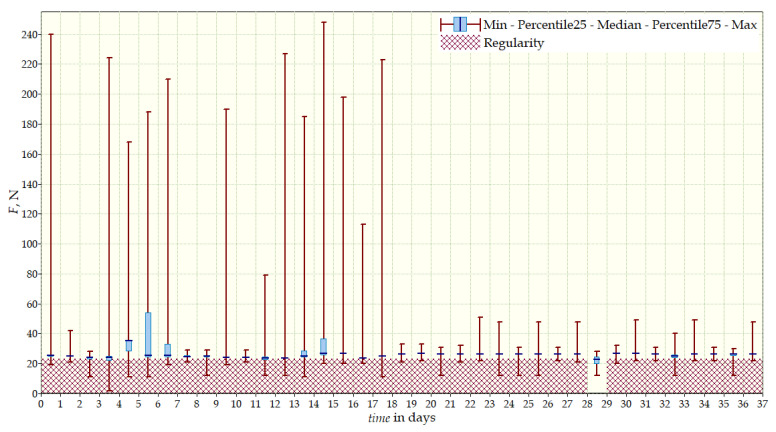
Measurement data from the second brace (two measurement periods)—sensor No. 1.

**Figure 19:**
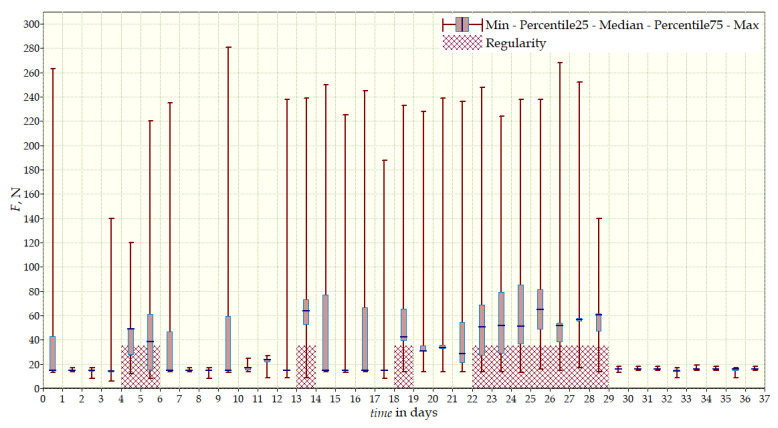
Measurement data from the second brace (two measurement periods)—sensor No. 2.

**Figure 20:**
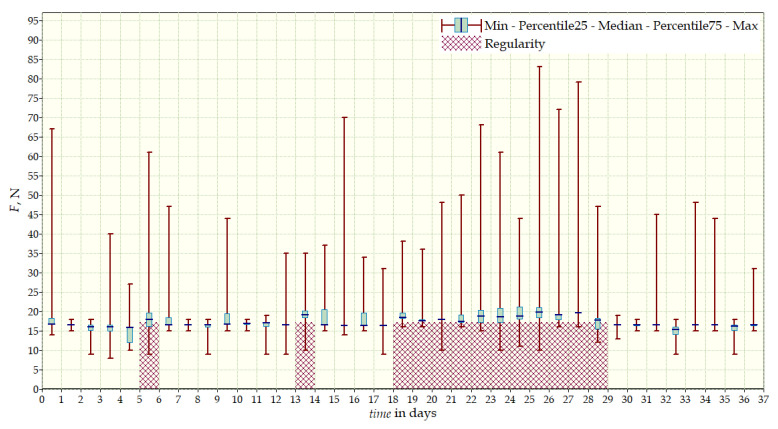
Measurement data from the second brace (two measurement periods)—sensor No. 3.

**Figure 21:**
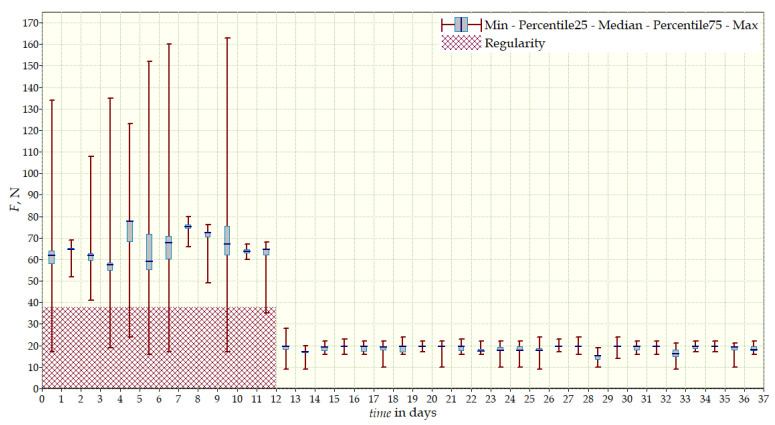
Measurement data from the second brace (two measurement periods)—sensor No. 4.

**Figure 22:**
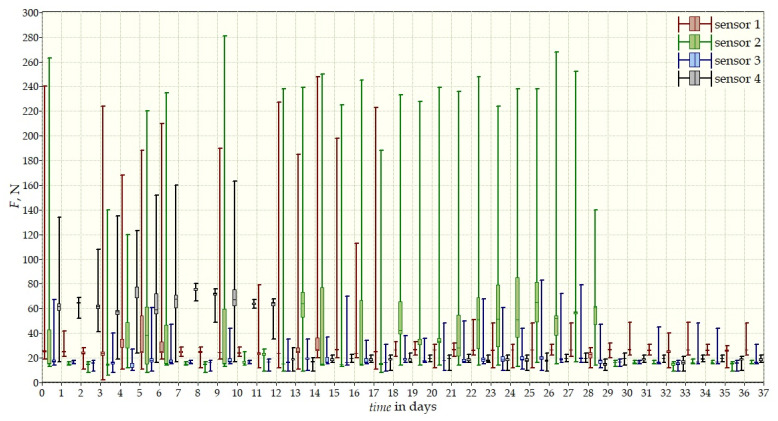
Measurement data from the second brace (two measurement periods).

The measurement data collected during the first patient trials were processed and visualized in graphs, showing the pressure forces changes in time. Figure 13, Figure 14, Figure 15 and Figure 16 show the measurement data, after averaging the measurements every 5 min from the first period of tests (the patient’s first orthosis, worn temporarily until the final brace was made), for each sensor separately, respectively. Figure 17 shows the data from all sensors on one graph. Figure 18, Figure 19, Figure 20, Figure 21 and Figure 22 show the measurement data from the second and third period of tests (in both, the second orthosis was used). The location of the individual sensors from the two braces was as follows:

1. orthosis (temporary):

- Sensor 1—on the chest, left side;
- Sensor 2—below the right shoulder blade, close to the spine;
- Sensor 3—below the right shoulder blade, to the right of sensor 2;
- Sensor 4—below the left shoulder blade.

2. orthosis (final):

- Sensor 1—below the right shoulder blade, to the right of sensor 2;
- Sensor 2—below the right shoulder blade, close to the spine;
- Sensor 3—on the chest, left side;
- Sensor 4—below the left shoulder blade.

The obtained data were processed and visualized, using the Lazarus environment, as box plots presenting changes in pressure forces as a function of time. The measured force ranged from 15 N to 250 N, depending on the sensor’s placement. Measurement data from the first period of trials (the patient’s first brace, worn temporarily until a proper brace was made), and data from the second brace from a longer measurement period, are presented.

After the data analysis, it was found that the samples do not have a normal distribution, therefore, the median and percentile values were used. The brace-wearing-regularity parameter shown on the graphs was developed on the basis of the data analysis from the entire period of wearing the brace. It allows the patient’s compliance with the therapy discipline to be determined. The large difference between the minimum and maximum values is caused by the physiological activities of the patient, e.g., breathing or physical activity. The graphs above show that the first (temporary) brace applied less pressure on the patient’s body, compared to the second (final) brace. The high values of the pressure forces on the sensors mounted on the patient’s back, especially on the sensor placed near the line of the spine, indicate significant activity of the orthosis in this region. Areas of unchanging pressure on any of the sensors are, likely, caused by not wearing the brace.

**Figure 23:**
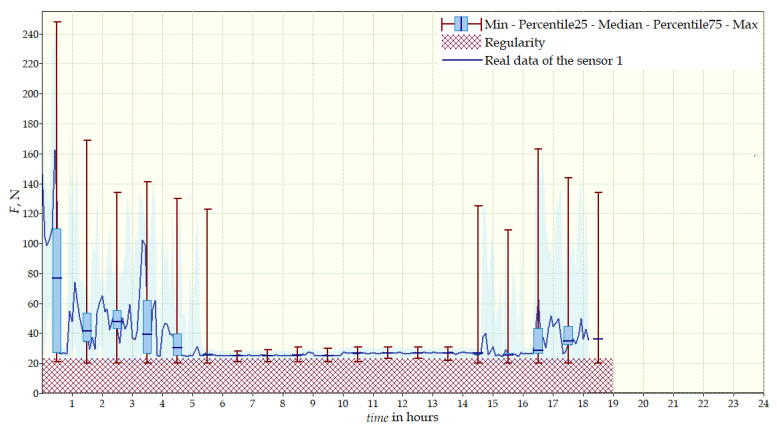
Measurement data for 24 h—sensor No. 1.

**Figure 24:**
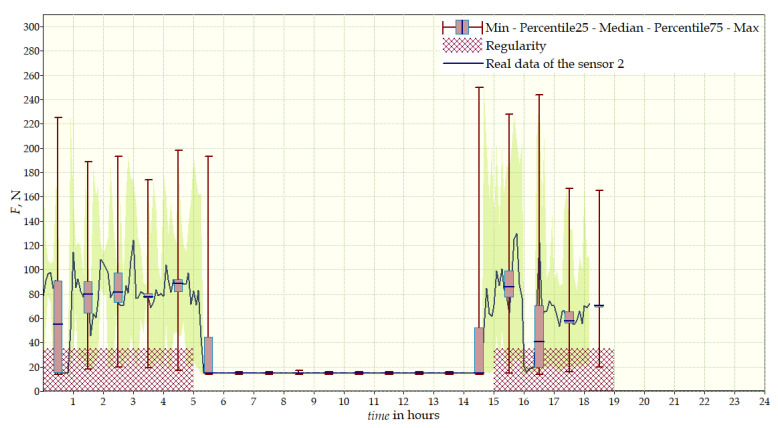
Measurement data for 24 h—sensor No. 2.

**Figure 25:**
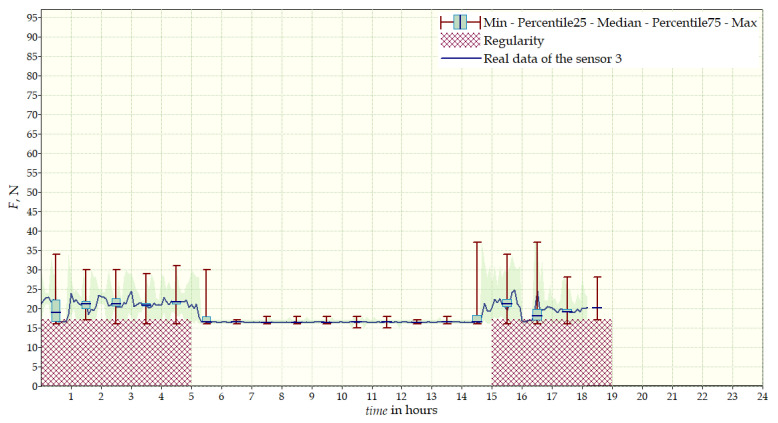
Measurement data for 24 h—sensor No. 3.

**Figure 26:**
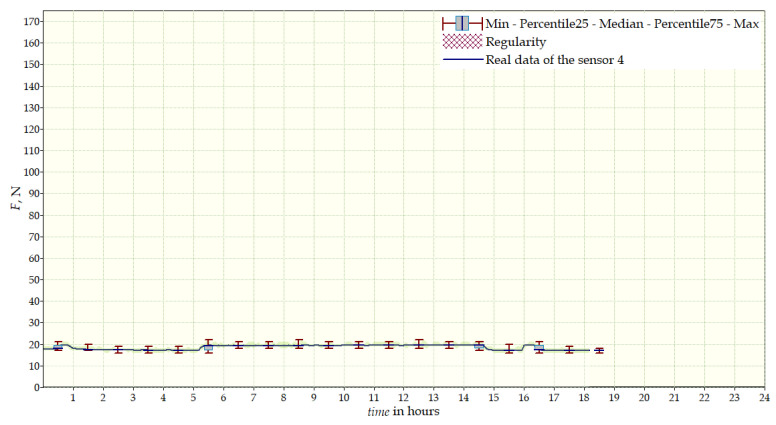
Measurement data for 24 h—sensor No. 4.

**Figure 27:**
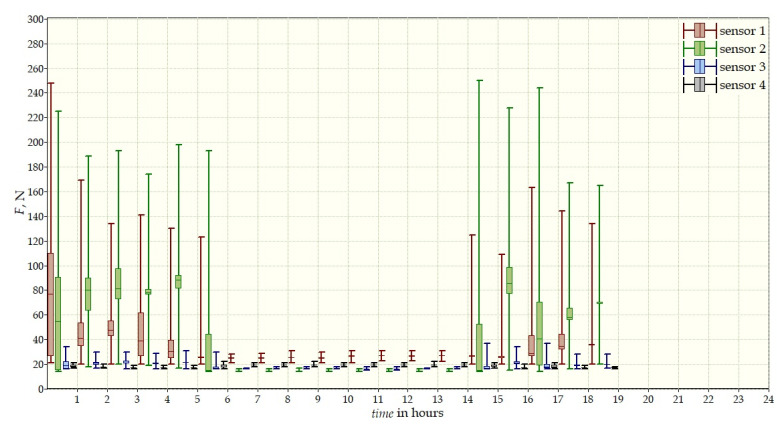
Measurement data for 24 h.

Data from a shorter period of time (Figure 23, Figure 24, Figure 25, Figure 26 and Figure 27) may confirm that the patient is not, systematically, wearing a brace, which, as mentioned earlier, will result in less-effective posture improvement. In these graphs, it is, also, possible to analyze fluctuations in the measurements from individual sensors, in more detail. Unchanging values on sensor no. 4 indicate its damage or disconnection from the system.

Despite many studies on the effectiveness and biomechanics of the orthopedic brace, there are no clearly defined ranges of therapeutic forces exerted by the brace, for specific types of curvatures in given age ranges. Experimental studies have attempted to measure both the pressure forces exerted by the brace on the body and the time the brace is worn [16]. The graphene sensors were designed to measure the effective pressure force (resulting in the intended correction of spinal curvature), exerted by the derotating pads in the brace, and to determine the reference range of applied pressures, depending on the patient’s bone age and type of curvature. The results will enable a more accurate prognosis of the therapeutic effects as well as the identification of new indications and recommendations, as to the adjustment of orthosis in individual age and angle groups. The research data obtained should be used in the future, to extend brace therapy to neurogenic and bone-derivative scoliosis. Despite the high therapeutic effectiveness of braces, there are, also, failures. The most frequent reason for a poor therapy effect is the patient’s lack of acceptance of long-term wearing of an uncomfortable orthosis [17] and an arbitrary shortening of therapy time. The fit of the brace, the appropriate pressure of the airpads, and the tension of the straps are, also, important [18]. Among the benefits of thin-film sensor application are: patient comfort (sensors are imperceptible by the patient), slight shadow on the X-ray image (no need to remove the sensors for radiological control), speed of operation, low element inertia, and no thermal impact. The location of sensor applications should be designed in the areas of highest derotating pads pressure. The collection of measurement data enables to build a statistical model of the brace’s influence on the patient’s torso, to learn the mechanisms of the brace’s dynamic influence in various functional positions (lying, sitting, walking). From the clinical point of view, the installation of sensors allows to eliminate design errors, improve the fit of the orthosis, and remotely control therapeutic discipline (compliance with recommendations). However, the application of sensors does not release the supervising doctor from the obligation to take an X-ray of the patient in the brace and evaluate the correction obtained.

## 5. Conclusions

The result of this work was a prototype brace that enabled data acquisition, using thin-film pressure sensors. The first trials on patients allowed to test the intelligent brace in real conditions and to verify the operation of the data acquisition system. It was, also, possible to find out what pressure forces are obtained by such a static brace. Errors in both the software and the hardware part of the system were detected, so we know the aspects in which the system needs to be optimized.

Having analyzed the data collected so far, there is a need to obtain more measurements, in order to create a decision-making system to control the pressure forces in the orthopedic brace. A bigger collection of measurement data will enable to build a statistical model of the brace’s influence on the patient’s torso and learn the mechanisms of the brace’s dynamic influence, in various functional positions (lying, sitting, walking). It should be noted that the brace biomechanics are optimized only for a standing posture. Hence, the interest of the research group towards determining the forces during the patient’s physical activity. There is a need to find out what the pressure force looks like in various body positions, such as sitting, lying, standing, and walking, as well as to be able to identify these positions and respond to them, adequately. The problem is the possibility of standardization, which, due to the individual therapeutic program, seems unjustified. Therefore, it is necessary to define the possibility of grouping and preparing data for the knowledge base, in such a way that it can be used in the decision-making system.

The desired result from this study is to show the potential of the described thin-layer pressure sensor solution, in the treatment of idiopathic scoliosis, by reducing the Cobb angle to correct spinal curvature.

## Abbreviations

The following abbreviations are used in this manuscript:

TLSO	Thoracic Lumbar Sacral Orthosis
IS	Idiopathic Scoliosis
DC	Direct Current
AC	Alternating Current
LCD	Liquid-Crystal Display
TFT	Thin-Film-Transistor
GSM	Global System for Mobile Communications
